# Management of liver disease patients in different clinical situations during COVID-19 pandemic

**DOI:** 10.1186/s43066-021-00091-x

**Published:** 2021-03-26

**Authors:** Samy Zaky, Mohamed Alboraie, Mohamed El Badry, Mohamed A. Metwally, Ahmed Abdelaziz, Yasser Fouad, Sherief Abd-Elsalam, Abdelmajeed Mahmoud, Gamal Shiha, Amin Abdel Baki, Mohamed El Kassas, Gamal Esmat

**Affiliations:** 1 Hepatogastroenterology and Infectious Diseases Department, Al-Azhar University, Cairo, Egypt; 2 Department of Internal Medicine, Al-Azhar University, Cairo, Egypt; 3 Endemic Medicine Department, Faculty of Medicine, Helwan University, 2-Ahmed Elzomor Street, Nasr City, Cairo Egypt; 4 Hepatology, Gastroenterology and Infectious Diseases Department, Benha University, Benha, Egypt; 5 Hepatogastroenterology and Infectious Diseases, Al-Azhar University, Demiatta, Egypt; 6 Tropical Medicine Department, Minia Faculty of Medicine, Minia University, Minia, Egypt; 7 Tropical Medicine Department, Tanta University, Tanta, Egypt; 8 Tropical Medicine and Gastroenterology Department, Aswan University, Aswan, Egypt; 9 Internal Medicine Department, Faculty of Medicine, Mansoura University, Mansoura, Egypt; 10 Department of Hepatology, National Hepatology and Tropical Medicine Research Institute, Cairo, Egypt; 11 Endemic Medicine and Hepatology Department, Faculty of Medicine, Cairo University, Cairo, Egypt

**Keywords:** SARS-CoV-2, COVID-19, Liver diseases, Hepatitis C, Hepatitis B

## Abstract

Chronic liver diseases are common worldwide, especially in developing countries. The rapid spread of severe acute respiratory syndrome coronavirus 2 (SARS-CoV-2)/(COVID-19) leads to the infection of many patients with underlying chronic liver diseases. As a relatively new disease, management of COVID-19, in the context of chronic liver disease, is mainly based on the experience of the treating physician and the available data. In this review, we summarize the available evidence about the management of liver disease patients, in the context of COVID-19 infection, which can increase the severity of viral hepatitis B. Also, its clearance in HBV patients is delayed. A sixfold increased severity of COVID-19 was reported in obese patients with metabolic associated fatty liver disease (MAFDL). In patients with autoimmune liver disease (AILD), it is not recommended to change their immunosuppressive therapy (as long as they are not infected with COVID-19), in order to avoid a flare of liver disease. However, immunosuppressant drugs should be modified, in the case of infection with COVID-19. To date, no data suggest an increased risk or severity in metabolic liver diseases, such as hemochromatosis, Wilson’s disease, or alpha-1 antitrypsin deficiency. Patients with liver cirrhosis should be carefully managed with minimum exposure to healthcare facilities. Basic investigations for follow-up can be scheduled at wider intervals; if patients need admission, this should be in COVID-19-clean areas. Patients with hepatocellular carcinomas may have a poor prognosis according to preliminary reports from China. The course of COVID-19 in liver transplant recipients on immunosuppression seems to have a benign course, based on few reports in children and adults. The hepatotoxicity of COVID-19 drugs ranges from mild liver enzyme elevation to a flare of underlying liver diseases. Therefore, the decision should be customized. Telemedicine can minimize the exposure of healthcare workers and patients to infection with COVID-19 and decrease the consumption of personal protective equipment.

## Background

The recently discovered coronavirus (COVID-19) caused by severe acute respiratory syndrome coronavirus 2 (SARS-CoV-2) has proven its pathogenicity to different organs including (but not limited to) respiratory, gastrointestinal, hematopoietic, renal, and cardiovascular systems [1–6]. Liver diseases have been reported to be associated with COVID-19 infection or secondary antiviral drugs used to control it [7]. Initial reports from China showed that liver enzyme derangements could occur in a considerable percentage (14–54%) of patients with COVID-19 infection [8]. The liver function test derangement was not limited to the elevation of aspartate aminotransferase (AST) and alanine aminotransferase (ALT) only. However, there was also evidence of abnormalities of alkaline phosphates, gamma-glutamyl transpeptidase, total bilirubin, albumin, and prothrombin time, which may reflect a direct effect of SARS-CoV-2 on hepatocytes [9]. Although most cases of liver enzyme derangement associated with COVID-19 can be transient and self-limited [8], cases of acute and chronic liver failure resulting from COVID-19 infection have been reported in patients with decompensated alcoholic and non-alcoholic liver cirrhosis [10, 11]. COVID-19 could lead to hepatorenal syndrome and may urge liver transplantation (LT) in infected patients with underlying advanced chronic liver disease (CLD) [11]. Patients with viral hepatitis, coinfected with COVID-19, need special care and individualization of treatment decisions. Treatment of newly diagnosed cases of hepatitis C virus (HCV) can be deferred until resolution of COVID-19 infection; however, chronic hepatitis B virus (HBV) patients may be offered treatment to prevent the flare of hepatitis B, which is frequently reported in cases of coinfection with COVID-19, especially if they were treated with tocilizumab or corticosteroid [12]. On the other hand, the presence of metabolic associated fatty liver disease (MAFLD) was an independent factor for the severity of COVID-19 in a series of non-diabetic patients infected with COVID-19, indicating that the relationship between liver disease and COVID-19 is bidirectionally detrimental [13].

## COVID-19 and viral hepatitis

There are relatively low screening rates for the most common viral hepatitis, HBV, and HCV in most diagnosed COVID-19 cases, especially in low and middle socioeconomic countries. Active screening is crucial for studying the clinical and epidemiological manifestations that could negatively affect public health efforts [14]. A 3% prevalence rate of having CLD in COVID-19 patients was observed in a meta-analysis from different reports from China, but this meta-analysis did not provide data on the prevalence of HBV and HCV infections in particular [15]. Despite that, HBV is common in China, where COVID-19 was first reported. The impact of COVID-19 infection on the clinical course of HCV and HBV is of great concern. It is worth, mentioning that COVID-19 has been infrequently reported in patients with either HBV or HCV infections in the USA. HBV and HCV infections were found in 0.1% and <0.1% of 5700 hospitalized COVID-19 patients from the northeastern USA, respectively [16]. On the other hand, HBV infection was found in 2.1% of 1099 hospitalized patients from Wuhan, China, and it represented 2.4% of non-severe cases and 0.6% of severe cases [17].

Moreover, a Chinese single-center retrospective study observed that 12.2% of 123 COVID-19 patients had HBV infection, and they developed a more severe course relative to HBV-negative patients and had higher total bilirubin levels (46.7% versus 24.1%); the mortality rate is 13.3% versus 2.8%, respectively [18]. Moreover, Zha et al. noted that the presence of COVID-19 infections on top of chronic HBV infection might delay COVID-19 clearance [19]. Some measures taken for COVID-19 prevention and control, such as social distancing and quarantine, can negatively affect the programs for diagnosis and treatment of HBV and HCV [20, 21]. As such, the treatment of viral hepatitis-infected patients can be affected by the closing of some private clinics and decreasing the number of working primary care clinics and general practitioners, who are now involved in the management of COVID-19 [19]. Therefore, increasing people’s awareness becomes the cornerstone in the elimination programs of viral hepatitis, as it provides more case finding and, hence, proper management.

Karimi-Sari and Rezaee-Zavareh noticed that hepatitis-related research articles published in PubMed, for example, during the COVID-19 era, are markedly decreased; this may clarify our idea about the impact of COVID-19 on the elimination programs of viral hepatitis [22]. Some reports suggested an approach for the treatment of patients with HBV or HCV during the era of COVID-19 either infected with COVID-19 or not based on the liver condition and the treatment experience [11]. Finally, there is no considerable published data regarding HEV or HAV and COVID-19. However, we appreciate systematic and integrated screening of HBV and HCV in COVID-19-confirmed patients; also, we advise developing relevant guidelines for management based on research, in order to understand the epidemiological pattern of viral hepatitis-COVID-19 coinfection.

## Metabolic associated fatty liver disease and COVID-19

A recent study reported that non-alcoholic fatty liver disease (NAFLD) was more prevalent in patients with severe compared with those with non-severeCOVID-19 illness [23]. MAFLD (metabolic dysfunction-associated fatty liver disease) recently replaced the term NAFLD [24]. The severity of MAFLD is determined by the severity of liver fibrosis [25, 26]. Nearly a 3-fold increased risk for severe COVID-19 was reported in obese patients with a direct relationship between increased BMI and the proportion of patients with severe illness [12]. Since obesity is an essential criterion in MAFLD patients, the risk of severe illness in MAFLD patients was highlighted in another study [27]. A 6-fold increased severity of COVID-19 was reported in obese patients with MAFLD. This increased risk was reported after adjustment for confounders. These findings are distinct, suggesting that the risk of COVID-19 severity, due to obesity, is significantly higher in those with MAFLD [2]. Interleukin-6 (IL-6), which is produced in large amounts in patients with severe COVID-19 and is considered a primary factor in triggering cytokine storm, is correlated with increased inflammatory activity (systemic inflammatory response, respiratory distress syndrome-induced hypoxia, or multiple organ dysfunction) in patients, particularly those with obesity [27, 28]. Another explanation was related to the reduced adiponectin or the altered secretion of inflammatory lipid mediators in obese patients with MAFLD [29].

In a cohort of 310 patients with confirmed COVID-19, a total of 94 patients were diagnosed with MAFLD. In this study, investigators found that patients with MAFLD with intermediate or high fibrosis 4 (FIB-4) scores were older and more obese and have a higher prevalence of diabetes, a higher level of liver enzymes, a higher level of C-reactive protein (CRP), and a lower level of lymphocyte count compared to patients without MAFLD or with low FIB-4 scores. Moreover, patients with MAFLD and increased scores of fibrosis were more likely to have severe COVID-19 illness, irrespective of the presence of metabolic abnormalities. In the same context, the presence of severe fibrosis in patients with MAFLD may exacerbate the virus-induced cytokine storm [30]. In another study on non-diabetic patients, MAFLD was associated with a fourfold increase in the severity of COVID-19 with adjusted age, gender, and other comorbidities [12].

The dysregulated hepatic innate immunity in patients with MAFLD may contribute to the pathogenesis of COVID-19. Abundant expression of ACE2 receptors was found in the enterocytes of the small intestine. This could be another portal of entry to COVID-19. In this context, one quarter of the COVID-19 patients are complaining of gastrointestinal manifestations, such as diarrhea and abdominal pain, without cough with positive COVID-19 RNA in fecal and respiratory specimens concomitantly [31]. Obesity and MAFLD are also associated with increased production of pro-inflammatory cytokines, such as TNF-α by adipose cells and Kupffer cells; this leads to the increased likelihood of symptomatic COVID-19 infections [32, 33]. Another study reported that MAFLD is not associated with changes in liver expression of genes implicated in COVID-19infection. Therefore, the elevated liver enzymes and deranged liver function tests in patients with MAFLD and COVID-19 cannot be explained by increased liver COVID-19 uptake and unlikely to be related to direct cytopathic effect [30]. Further studies are needed to enhance our understanding of the link between the dysregulated hepatic innate immunity and COVID-19.

## Autoimmune liver disease

Autoimmune liver disease (AILD) is a group of diseases that include autoimmune hepatitis (AIH), primary biliary cholangitis (PBC), primary sclerosing cholangitis (PSC), and overlap syndrome. Although the diagnosis of AIH is based on histological abnormalities (interface hepatitis), characteristic of clinical and laboratory findings (elevated AST and ALT levels and increased serum IgG concentration), and the presence of one or more characteristic autoantibodies [34–36], the diagnosis of new AIH cases and starting medication strategy during the COVID pandemic could be subject to change. Histopathological characteristics that necessitate hospital visits and liver biopsy, which could constitute a risk of COVID-19 infection for those patients, can be waived and only depending on high clinical and laboratory precautions for starting empirical treatment with a corticosteroid. Confirmation of the diagnosis can be done by liver biopsy, after the regression of COVID-19 pandemic, or by the presence of relapse during the withdrawal from treatment [37]. Although COVID-19 infection is associated with ALT and AST elevation in 14–53% of the population, especially in hospitalized patients, most of whom have mild elevation up to twofolds. In rare occasions, some patients may have severe hepatitis [7, 16, 38, 39]. Thus, COVID-19 infection should also be considered, when evaluating patients with elevated ALT and AST. When there is a high indication of immunosuppression, as in the case of AIH, the medication should start, regardless of the patient is infected or not infected with COVID-19. The data from previous studies about the mortality of patients on immunosuppressive therapy, who had been infected with other coronaviruses (i.e., severe acute respiratory syndrome coronavirus (SARS-CoV), or Middle East respiratory syndrome coronavirus (MERS-CoV)), showed that the case fatalities in patients who were undergoing transplantation, chemotherapy, or other immunosuppressive treatments, at any age, were not different from the general population [40]. Data from Bergamo in Northern Italy with a high COVID-19 infection rate showed that patients with AILD on immunosuppressive therapy have a similar rate of infection and case fatality, as found in the general population in the same area [2]. Immunosuppressive medication may, even, have a protective effect against severe COVID-19 infection complications, which are suspected to occur due to the cytokine storm [41]. That is why the European Association for the Study of the Liver and the American Association for the Study of Liver Diseases (EASL and AASLD) came against reducing immunosuppressive drugs for AILD patients, if they have not been infected with COVID-19. Additional reduction of immunosuppressive therapy may lead to more risky conditions with the flare-up of hepatitis that necessitates higher doses of corticosteroid with more susceptibility to infection [1, 42]. Data about patients with AILD on immunosuppressive therapy, who have been infected with COVID-19, are limited. Other healthcare organizations, such as the Centers for Disease Control and Prevention (CDC), considered patients on immunosuppressive therapy to be at higher risk for severe illness from COVID-19 and have prolonged the shedding of the virus [43]. Although there are no clear guidelines regarding the management of immunosuppressive therapy in AILD patients infected with COVID-19, the rule of thumb is no reduction of immunosuppressive therapy unless a confirmed infection, fever, and lymphopenia are found. The AASLD recommendations were as follows: in immunosuppressed liver disease patients with COVID-19, consider the following:

- Minimizing the dosage of high-dose prednisone but maintaining a sufficient dosage to avoid adrenal insufficiency.
- Reducing azathioprine or mycophenolate dosages, especially in the setting of lymphopenia, fever, or worsening pneumonia attributed to COVID-19.
- Reducing, but not stopping, daily calcineurin inhibitor dosage, especially in the setting of lymphopenia, fever, or worsening pulmonary status attributed to COVID-19 [41].

A more detailed recommendation report about the management of patients on immunosuppressive medication was published by the Beijing Working Team for liver transplantation. Although it is targeting liver-transplanted patients, it can be applicable to AILD patients on immunosuppressive therapy. The recommendations were as follows:

- No need to adjust the dose of immunosuppressive drugs in patients without COVID-19.
- For patients with mild to moderate COVID-19, keep current immunosuppressant dosage and closely monitor the patient’s condition. Avoid prescription of medications that cause significant fluctuations in tacrolimus plasma concentrations.
- For patients with severe or rapidly progressing COVID-19, reduce the amount of calcineurin inhibitor and consider stopping anti-metabolic drugs as mycophenolate and azathioprine.
- If decided to reduce the dose of steroids, keep at least a daily dose of 10 mg prednisone or equivalence to avoid adrenal insufficiency.
- Corticosteroids or other immunosuppressive therapies can be re-initiated with caution when the potential benefits outweigh their discontinuation [44].

Patients with AILD, who have cirrhosis or one of it is complications, should be treated according to the recommendation of treatment of complications of cirrhosis during the COVID-19 epidemic [45].

## Metabolic liver diseases

### Hemochromatosis

In light of previous evidence, people with genetic hemochromatosis are not at increased risk of contracting COVID-19 infection. So, the only risk of infection depends on the patient’s behavior with the best protective measures being to stay home and practice social distancing. However, the most critical question is whether this particular category of patients will be at high risk of complications, if they contract COVID-19 infection [1, 46], (https://www.haemochromatosis.org.uk/cornonavirus-update-march-2020).

As COVID-19 is a novel virus, its interactions with people with genetic hemochromatosis have not been specifically studied; however, people who are at increased risk of complications are those with advanced age or with cardiac, liver, or comorbid disease. Theoretically speaking, this special category of patients, if they have diabetes, a heart condition, or liver cirrhosis, is at increased risk of complications [47–49].

Venesection must be delayed if the patient contracts coronavirus infection or is self-isolated; in this case, the patient has to avoid attending to his general practitioner or the hospital or even to blood donation centers to avoid transmission of infection to others, including the medical teams. If the patient is in good health, he can attend his session of venesection unless he has been instructed by the local health authority not to attend (https://www.haemochromatosis.org.uk/cornonavirus-update-march-2020), [50].

### Wilson disease

Wilson’s disease does not appear to increase the risk of contracting COVID-19. There is a common belief between scientists, with insufficient evidence, that patients with well-controlled Wilson disease are not highly susceptible to COVID-19 than the general population. As COVID-19 is a novel virus, its interactions with people with Wilson disease have not been specifically studied; however, people at increased risk of complications are those with advanced age, severe liver, heart disease, and renal failure or comorbid disease. Therefore, theoretically speaking, this special category of patients, if they have liver cirrhosis or severe comorbid disease, is at increased risk of complications [45, 51–53].

Patients with Wilson disease are strictly advised not to stop their medications to avoid exacerbation of the disease. Dose adjustment may be considered, if copper control is excellent and low copper status causes a decrease of white blood cells after consultation with the treating physician [51, 53].

### Alpha-1 antitrypsin deficiency

Since COVID-19 primarily causes fever and respiratory symptoms and often leads to pneumonia in exposed people, people with alpha-1 are more likely to be particularly vulnerable to coronavirus infection. Alpha-1 deficiency affected lung disease, or those who have had a lung transplant are among the groups that are most at risk of severe complications, if they become infected [54]. This particular category of vulnerable patients should strictly follow the preventative actions to stay home, practice social distancing, and avoid contact with patients with respiratory illness [53]. Finally, for those with Alpha-1 who are treated with augmentation treatment, the Plasma Protein Therapeutics Association (PPTA) stated that there was no risk of transmission of COVID-19 through injection of the plasma product. To date, there have been no delays in augmentation treatment supply, due to COVID-19 [54].

### Liver cirrhosis

The following guidelines were recommended for the management of patients with liver cirrhosis, during the COVID-19 pandemic, by the latest European Association for the Study of the Liver (EASL) and the European Society of Clinical Microbiology and Infectious Diseases (ESCMID) position paper [55].

## For patients without COVID-19

Outpatient care:

- Visits to specialized liver centers can be postponed, and these centers should provide accessible contact information for local healthcare providers to facilitate good co-operation in the management of patients with liver cirrhosis.
- Use telemedicine consultations, by phone, wherever possible.
- Routine follow-up laboratory testing can be performed locally, e.g., through primary care physicians; its frequency needs careful individual risk-benefit considerations.
- Hepatocellular carcinoma (HCC) surveillance: abdominal ultrasound examination can be deferred based on available resources (including the availability of therapeutic options in case of HCC diagnosis), at the center and the individual risk assessment. Patients with increased risk, such as patients with elevated alpha-fetoprotein levels and advanced cirrhosis with chronic hepatitis B virus infection, may be prioritized if resources are limited [44].
- Screening for varices: depending on available resources, screening for varices by esophagogastroduodenoscopy (EGD) should be reserved for patients at risk of variceal bleeding, such as patients with a history of variceal bleeding or signs of significant portal hypertension (ascites, splenomegaly, and platelet count <100,000/μl). Non-invasive risk assessment for the presence of varices can be used to stratify the portal hypertensive status of the patients [56].
- Guidelines on prophylaxis of spontaneous bacterial peritonitis and hepatic encephalopathy, in patients with decompensated cirrhosis, should be carefully followed to prevent decompensation and avoid hospital admission during this pandemic.
- Include testing for SARS-CoV-2 in any patients with acute decompensation of their liver status or patients with acute on top of chronic liver cell failure.
- Emphasis on the importance of vaccination of the patients for streptococcus pneumonia and influenza.

Inpatient care:

- Many patients will require inpatient care during the COVID-19 pandemic for decompensation or other complications like variceal bleeding, spontaneous bacterial peritonitis, and hepatic encephalopathy. General measures to prevent SARS-CoV-2 exposure and infection will be of utmost importance for these patients. Depending on the local infrastructure, the implementation of COVID-19-clean wards or hospitals is warranted. Wherever possible, patients with liver cirrhosis requiring inpatient care for non-COVID-19 causes should be admitted to COVID-19-clean wards or hospitals [44].

## For patients with COVID-19

General considerations:

- Consider early admission according to the presence of additional risk factors and inclusion in clinical trials and (experimental) antiviral therapy of COVID-19 following local guidelines.
- Treatment for cirrhosis-associated complications, such as portal hypertension, ascites, hepatic encephalopathy, and spontaneous bacterial peritonitis, should be continued.
- Avoid acetaminophen overdosing (2–3 g/day is considered safe in patients with cirrhosis, without current alcohol consumption) and avoid the use of non-steroidalanti-inflammatory drugs in patients with cirrhosis and portal hypertension [57].
- Anticoagulants can be used in patients with liver cirrhosis in the absence of absolute contraindications, keeping in mind the status of varices in this group of patients before starting anticoagulants [58].
- Endoscopic procedures in patients with COVID-19 should be limited to emergencies, such as gastrointestinal variceal bleeding.
- HCC surveillance should be deferred until after recovery from COVID-19.

Specific treatment considerations:

Although there are currently no drugs approved for COVID-19, several repurposed drugs have been tested in recent weeks, and many are still under investigation [59]. However, the use of these drugs in patients with liver cirrhosis had possible potential adverse events.

- Chloroquine/hydroxychloroquine: these drugs are not usually associated with hepatotoxicity and an infrequent cause of clinically significant acute liver injury [60].
- Lopinavir/ritonavir: not recommended to be used in patients with decompensated liver cirrhosis [61].
- Favipiravir/favilavir: no data available on their use in patients with liver cirrhosis.
- Remdesivir: no data available on its use in patients with liver cirrhosis. Reportedly, it can cause raised liver enzymes [62].
- Tocilizumab: reportedly, it could cause raised liver enzymes, rarely linked to severe liver injury, and better not to be used in patients with decompensated cirrhosis [63].
- Convalescent plasma: no data available on its use in patients with liver cirrhosis.

## Management of hepatocellular carcinoma in the time of COVID-19

HCC represents the sixth most common cancer worldwide [44]. HCC surveillance is associated with both early tumor detection and improved survival [44, 64]. Patients with HCC are, theoretically, at increased risk for severe COVID-19, either due to malignancy or treatment. COVID-19 was reported in 28 cancer patients from China. Two of them (7%) with HCC are at high risk of poor outcomes in comparison with non-infected patients according to age, comorbidities, and underlying cirrhosis [64]. Moreover, patients with non-hepatic malignancies who underwent recent chemotherapy were associated with worse COVID-19 outcomes [65]. Currently, clinical activities are being reduced to decrease the risk of transmission and to reserve healthcare workers for facing the pandemic. However, it is accepted that more intensive attention should be paid to patients with cancer during the COVID-19 crisis, both for reducing the risk of severe acute respiratory syndrome-relatedCOVID-19 infection and ensuring appropriate cancer patient management programs.

The following are the recommendations regarding HCC management during the COVID-19 pandemic;

- ➢ Surveillance should be continued for those at risk (cirrhosis, chronic HBV) as scheduled, although a 2-month delay may be reasonable [44].
- ➢ The multidisciplinary meetings are conducted by conference calls to reduce the risk for healthcare operators without delaying decisions [66].
- ➢ Proceed with liver cancer treatments or surgical resection, whenever possible, rather than delaying them due to the pandemic [67].
- ➢ Locoregional treatment (LRT) is preferred when applicable, and surgery should be left only as a salvage option for those who fail to complete radiological response or are not appropriate for LRTs [44].
- ➢ LT should be reserved for patients with a high risk of dropout, due to the shortage of intensive care unit bed availability and the dramatic reduction in the donor pool; LRT, whenever possible, may be used as a bridge treatment to reduce the risk of disease progression, during the waiting period [34].
- ➢ Palliative treatments, such as trans-arterial chemo (radio) embolization (TACE, TARE), are maintained; however, in old ages (>80 years) or patients with comorbidities, palliative should be delayed, weighing the oncological benefit versus the risk of exposure to SARS-CoV-2 [66].
- ➢ Imaging techniques carried out for diagnosis, staging, or follow-up should be revised via telemedicine. Radiological follow-up is scheduled as usual, but postponed up to 3 months, in elderly patients and those with comorbidities [66].
- ➢ The COVID-19 test is carried out in all patients 1 day before admission; only negative patients are admitted to dedicated rooms in the ward (to protect them against nosocomial COVID-19 infection) [54].

### COVID-19 and liver transplantation

LT remains the only curative treatment for selected patients with end-stage liver diseases and acute liver failure. Survival rates have improved significantly in the last 25 years [68]. Advances in surgical techniques, the introduction of new immunosuppressive agents, and the early diagnosis and management of complications after LT are the main factors contributing to such great success [69]. COVID-19 virus can remain viable and infectious in aerosols for hours and on surfaces up to days (depending on the inoculum shed), which raises the concern of nosocomial spread [70]. Moreover, patient-to-patient and patient-to-healthcare worker infections have been described. It is unknown whether COVID-19 can be transmitted parenterally or not; nevertheless, screening of all blood or organ donors is recommended [71]. Theoretically, liver transplanted patients may have a more significant viral burden and prolonged shedding of the virus resulting in higher infectivity and potential spread to other individuals, including healthcare workers. There is a risk of the donor to recipient transmission of COVID-19 [34]. The impact of immunosuppression in the post-transplant setting is currently unknown; however, there is a concern that immunocompromised patients are at a higher risk of morbidity and mortality due to COVID-19 infection, although data on COVID-19 in liver transplant patients are scarce [34, 71]. A case series, from Italy, showed that children who had received liver transplants, despite being immunosuppressed, were not at increased risk of severe pulmonary disease than the general population suggesting that immunosuppression may protect against cytokine storm induced by COVID-19, which is responsible for the severe illness [72]. More over, three of 111 long-term liver transplant survivors (transplanted more than 10 years ago) have died following severe COVID-19 disease. All three were male, older than 65 years, receiving antihypertensive drugs, overweight (BMI >28 kg/m^2^), with hyperlipidemia and diabetes (median HbA1c of 6.9%), and their immunosuppressive regimen had been gradually tapered off, with deficient concentrations of calcineurin inhibitors. By contrast, three of 40 recently transplanted (i.e., within the past 2 years) patients have tested COVID-19-positive, and although quarantined, all are experiencing an uneventful course of disease [73]. It was noticed that, despite concerns that liver transplant (LT) recipients may be at high risk of unfavorable outcomes from COVID-19 due to the high prevalence of co-morbidities, immunosuppression, and aging, a detailed analysis of their effects in large studies is lacking [74]. Other challenges also need to be considered: the dramatic reduction in the donor pool during the pandemic, risk of infection on the healthy donor with hospital admission, the scant ICU beds available, and the drug-drug interactions between immunosuppressive medications and COVID-19 therapy [43].

### Recommendations

A- Before liver transplantation
  ➢ All donors (deceased and living donors) and recipients must be tested for COVID-19 at the time of urgent transplant using nasopharyngeal swab in approved laboratories [70]. Moreover, a rapid test for the transplantation team is highly recommended [74].
  ➢ A detailed history regarding exposure history, fever, and respiratory symptoms should be reviewed. Additional data including chest imaging and inflammatory markers (e.g., C-reactive protein, ferritin, IL-6) should be considered to minimize the significant false-negative testing rate [75].
  ➢ COVID-19-positive transplant candidates may be considered for transplantation at least 14–21 days after symptom resolution and 1 or 2 negative COVID-19 diagnostic tests [75].
  ➢ Preventing transmission from an infected patient to a healthcare worker and vice versa is of great importance. Current recommendations are for both droplet and airborne precautions for infection control, in the hospital setting. Social distancing by widening the patients’ waiting areas, frequent hand washing, cleaning frequently touched surfaces, following cough etiquettes, and strict infection control precautions are critical [34, 70].
  ➢ Consider evaluating only patients with HCC or those patients with high Model for End-Stage Liver Disease (MELD) scores, who are likely to benefit from immediate liver transplant listing. Telemedicine can be used for evaluation, whenever applicable [75].
  ➢ Consider resource utilization, including intensive care unit (ICU) beds, ventilators, personal protective equipment (PPE), and supply of blood products in the decision to proceed with liver transplantation [34, 75].
  ➢ Moratorium on all non-urgent transplants. Liver transplantation should be restricted only to those with high MELD scores and HCC patients [70]. LRT, whenever possible, should be used as bridge treatment in patients with HCC [44].
  ➢ Pre-procedure consent should include the potential hazard for the acquisition of nosocomial COVID-19 infection [54].
B- During the procedure
  ➢ Strict infection control precautions and the use of (PPE) for all surgical teams are essential [76].
C- Post-operative care for liver transplant patients.
  ➢ Liver transplant recipients should be admitted to separate wards, with strict implementation of prevention measures [77].
  ➢ Limit or even prohibit non-essential team members in the hospital (e.g., students, observers, research staff) to minimize exposure risk and prioritize the use of PPE [71].
  ➢ Minimize in-person visits for post-transplant patients, by maximizing the use of telemedicine. However, those patients with post-transplant emergencies should attend hospital, as usual, and should be provided with necessary standard care by the transplant team [71].
  ➢ Limit the number of visitors who may see inpatients. Ideally, no visitors should be permitted in patient rooms, except in specific circumstances (e.g., hospice care, pediatrics, a patient being discharged following transplantation) [34, 71].

### Regarding immunosuppressive therapy

1. In post-transplant patients without COVID-19:
  There is no evidence, until now, for the need to modify the immunosuppression protocol. Standard immunosuppression should be followed in the post-transplant period, until further data is available [71].
2. In post-transplant patients with COVID-19:
  The National Institute of Health (NIH) recommends against the routine use of systemic corticosteroids for the treatment of COVID-19 in hospitalized patients, unless they are in the intensive care unit [78]. Minimize the dose of prednisone, but maintain a sufficient dosage to avoid adrenal insufficiency [74].
  Consider reducing azathioprine or mycophenolate dosages, especially in the setting of lymphopenia, fever, or worsening pneumonia attributed to COVID-19 [34].
  Consider reducing, but not stopping daily calcineurin inhibitor dosage, especially in the setting of lymphopenia, fever, or worsening pulmonary status attributed to COVID-19 [74].

### Potential telemedicine applications for care of liver disease patients

Telehealth (or telemedicine) service implies providing medical care at a distance where both the patient and his healthcare worker are in geographically distant places. Providing regular face-to-face medical services in clinics and hospitals has been significantly replaced by telehealth services during the pandemic of COVID-19 [79]. In the hepatology field, telemedicine has been used successfully in treating hepatitis C patients, even before the SARS-CoV-2 pandemic. Extension for Community Healthcare Outcomes (ECHO) project enrolled chronic hepatitis C patients in underserved communities (rural areas or prisons) and treated them, with the help of primary care physicians assisted by hepatologists, from New Mexico University, through videoconference calls [80]. This model has proven to be successful in different parts of the world [81]. Outpatient follow-up, for stable CLD patients (or compensated cirrhotic), can be scheduled at a longer time with interim visits and laboratory evaluations carried by their local primary care physician, who can consult hepatologists, if needed, through telemedicine [54]. This may decrease the risks for liver disease progression and non-compliance to regular clinic visits for those patients [82]. Telemedicine can also be applied in a liver transplant setting, allowing faster evaluation and shorter time to put the patients on the waiting list [83]. Telemedicine can minimize the exposure of healthcare workers and patients to infection with COVID-19 and decrease the consumption of personal protective equipment. However, there is a limitation in preforming formal clinical examination and difficulty to use by some patients [78, 84].

### COVID-19 treatment and potential hepatotoxicity

In patients with chronic liver disease, possible adverse events have to be considered with the use of COVID-19 treatment regimens [54]. Remdesivir is a nucleoside analog (NUC) that is approved in the USA for emergency use for COVID-19, which acts as a viral RNA polymerase inhibitor. It inhibits SARS-CoV-2 in vitro [85] as well as in case reports of patients with COVID-19 [86]. A double-blindplacebo-controlled randomized study in 1063 patients confirmed beneficial findings of case reports [87]. There is no experience, so far, in patients with liver cirrhosis. However, based on experience with NUCs in chronic hepatitis B and C, it might be considered as a safer drug than other drug classes [54]. Liver toxicity with ALT elevation is a possible adverse event. No relevant drug-drug interactions are expected [88]. Other drugs currently under evaluation include chloroquine and hydroxychloroquine that interfere with the cellular receptor ACE2 and act as an endosomal acidification fusion inhibitor. These drugs have long been used for the treatment of malaria, amoebiasis, and autoimmune conditions. Hepatotoxicity, due to hydroxychloroquine, has been described, but it is rare [67]. Lopinavir/ritonavir is approved protease inhibitors (PIs) for second-line HIV treatment. Many centers have discontinued their experimental use, as there is no proven efficacy of in vivo, in severe COVID-19 [60]. Patients with decompensated cirrhosis should not be treated, based on the experience with protease inhibitors in HCV [62]. Tocilizumab is a humanized monoclonal antibody against the interleukin-6 receptor that works by damping the cytokine release syndrome observed in COVID-19 patients. Liver toxicity in the form of ALT elevations is frequent, but clinically apparent liver injury with jaundice seems to be rare [89]. HBV reactivation is a considerable adverse event in the case of its administration [90]. Moreover, it should not be used in patients with decompensated cirrhosis [54]. In randomized clinical trials, convalescent plasma did not show clinical benefit, reduce progression to severe COVID-19 or improve the overall mortality [91, 92]. There is no experience with convalescent plasma therapy in COVID-19 patients with chronic liver disease [54]. Favipiravir is a guanine analog, that is an RNA-dependent RNA polymerase (RdRp) inhibitor, approved for influenza in Japan. Studies have revealed that favipiravir showed better treatment outcomes in COVID-19 patients in terms of their disease progression and viral clearance [92]. ALT and AST elevation is a possible side effect, while no data in cirrhosis are available [54].

## Conclusion

Management of chronic liver disease during the COVID-19 pandemic is challenging. Several patients with underlying chronic liver disease are prone to the rapidly spreading SARS-CoV-2 infection, which can affect their underlying liver disease. Patients with hepatitis B, MAFLD, autoimmune liver disease, and HCC and liver transplant recipients need special attention for follow-up and treatment management. Hepatotoxicity of COVID-19 medications, if managed properly, can be overcome. Telemedicine can help in decreasing contact between patients and potential sources of infection.

## Data Availability

Data is available upon request.

## Abbreviations

ALT: Alanine aminotransferase
AST: Aspartate aminotransferase
AIH: Autoimmune hepatitis
AILD: Autoimmune liver diseases
HBV: Chronic hepatitis B virus
CLD: Chronic liver disease
COVID-19: Coronavirus
EGD: Esophagogastroduodenoscopy
EASL: European Association for the Study of the Liver
ELTR: European Liver Transplant Registry
ESCMID: European Society of Clinical Microbiology and Infectious Diseases
HAV: Hepatitis A virus
HCV: Hepatitis C virus
HEV: Hepatitis E virus
HCC: Hepatocellular carcinoma
LRT: Locoregional treatment
MAFLD: Metabolic associated fatty liver disease
MERS-Cov: Middle East respiratory syndrome coronavirus
NUC: Nucleoside analog
PPE: Personal protection equipment
PPTA: Plasma Protein Therapeutics Association
PBC: Primary biliary cholangitis
PSC: Primary sclerosing cholangitis
PIs: Protease inhibitors
Rdrp: RNA-dependent RNA polymerase
SARS-Cov-2: Severe acute respiratory syndrome coronavirus 2
TACE, TARE: Trans-arterial chemo(radio) embolization
